# N^6^-methyladenosine (m^6^A) RNA modification in chronic myeloid leukemia: unveiling a novel therapeutic target

**DOI:** 10.1007/s00018-024-05379-w

**Published:** 2024-07-31

**Authors:** Guillermo Fernandez Rodriguez, Marco Tarullo, Alessandro Fatica

**Affiliations:** Department of Biology and Biotechnologies “Charles Darwin”, Sapienza University of Rome, 00185 Rome, Italy

**Keywords:** m^6^A, CML, Epitranscriptomics, Cancer

## Abstract

N^6^-methyladenosine (m^6^A), the most prevalent internal mRNA modification, plays a critical role in physiological processes by regulating gene expression through modulation of mRNA metabolism at multiple stages. In recent years, m^6^A has garnered significant attention for a deeper understanding of the initiation, progression, and drug resistance of various cancers, including hematological malignancies. Dysregulation of m^6^A has been implicated in both cancer promotion and suppression. m^6^A methylation is a complex regulatory process involving methyltransferases (writers), demethylases (erasers), and proteins that recognize specific m^6^A modifications (readers). This intricate interplay presents challenges for precisely modulating m^6^A levels, either globally or at specific sites. This review specifically focuses on the role of m^6^A in chronic myeloid leukemia (CML), a blood cancer characterized by the BCR-ABL1 fusion. We emphasize its impact on leukemia cell survival and drug resistance mechanisms. Notably, inhibitors targeting m^6^A regulators show promise in preclinical models, suggesting a potential therapeutic avenue for CML. Integrating our understanding of m^6^A biology with current treatment strategies may lead to more effective therapies, especially for patients with advanced-stage or resistant CML.

## Introduction

**Fig. 1:**
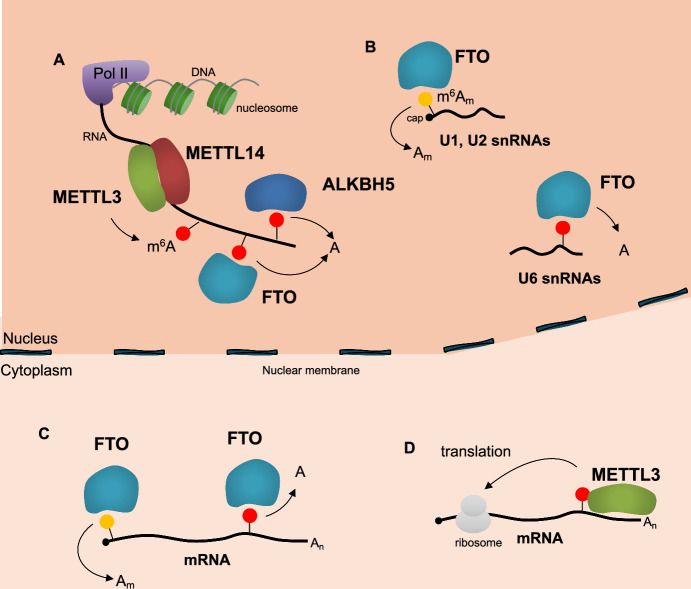
Writers and erasers of m^6^A modification. m^6^A modification within RNA molecules is dynamically regulated by a balance between "writer" methyltransferase complexes and "eraser" demethylase enzymes. **A** In the nucleus, the METTL3-METTL14 complex acts as the primary writer, modifying adenines co-transcriptionally onto mRNA. Conversely, two demethylases, ALKBH5 and FTO, can remove m^6^A from nuclear RNA substrates. **B** Notably, nuclear FTO exhibits broader substrate specificity, demethylating internal m^6^A within U6 small nuclear RNA (snRNA) and m^6^A_m_ in the cap region of U1 and U2 snRNAs. **C** Additionally, cytoplasmic FTO contributes to m^6^A regulation by demethylating both m^6^A_m_ in the mRNA cap and internal m^6^A modifications within mature mRNAs. **D** Furthermore, METTL3 can indirectly promote translation by directly binding to mRNAs in the cytoplasm. Across all panels, DNA is depicted as a grey line, the nucleosome as a green barrel, RNA as a black line, the 5′ cap structure as a black dot, the poly(A) tail as A_n_, the m^6^A modification as a red circle, and the m^6^A_m_ modification as a yellow circle.

**Fig. 2:**
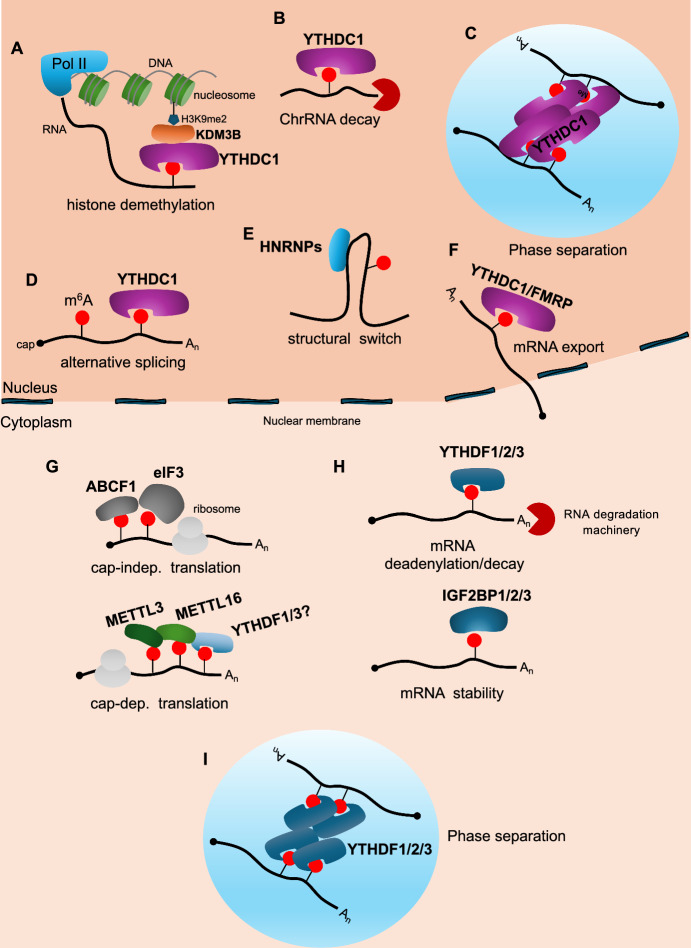
Modulation of mRNA function by m^6^A modification. m^6^A modifications on RNA molecules exert widespread effects on various cellular processes. m^6^A reader proteins specifically recognize and bind to this mark, dictating the functional consequences of m^6^A on RNA expression. Within the nucleus, m^6^A can influence gene regulation through diverse mechanisms: **A** regulation of H3K9 Methylation, the YTHDC1 reader can influence transcription by regulating the methylation state of histone H3 at lysine 9 (H3K9); **B** Chromatin-Associated RNA (chrRNA) Targeting, YTHDC1 binds to and mediates the degradation of chrRNAs, thereby impacting chromatin structure and function; **C** nuclear mRNA condensates and stability, YTHDC1 can directly bind specific mRNAs and promote their liquid–liquid phase separation into condensates, potentially enhancing their stability; **D** alternative splicing regulation, the presence of m^6^A in pre-mRNAs can influence alternative splicing events by recruiting YTHDC1; **E** m^6^A switch, m^6^A can induce conformational changes in RNA structure, leading to altered binding of regulatory proteins and affecting splicing or other RNA processing events; **F** mRNA export, YTHDC1, along with other factors like FMRP, can stimulate the export of mature mRNAs from the nucleus to the cytoplasm. Within the cytoplasm, m^6^A modifications exert a multifaceted influence on mRNA metabolism. **G** The position of the m^6^A mark on the mRNA dictates its impact on translation. m^6^A located in the 5′-UTR can influence cap-independent translation. The ABCF1 and eIF3 readers can bind m^6^A present in the 5′-UTR of mRNAs and stimulate translation initiation, while the YTHDF1 and YTHDF3 readers can stimulate cap-dependent translation by binding m^6^A in the 3′ regions of mRNA. However, recent research has yielded conflicting results regarding the involvement of YTHDF1 and YTHDF3 in regulating translation. Furthermore, cytoplasmic METTL3 and METTL16 can regulate cap-dependent translation by a catalytic-independent mechanism. **H** mRNA stability**,** YTHDF1/2/3 readers can trigger mRNA decay pathways when they bind to m^6^A. Conversely, IGF2BP1/2/3 can enhance the stability of mRNAs containing m^6^A modifications.** I** Finally, YTHDFs readers can promote liquid–liquid phase separation into condensates of m^6^A-modified mRNAs. Across all panels, DNA is depicted as a grey line, the nucleosome as a green barrel, RNA as a black line, the 5' cap structure as a black dot, the poly(A) tail as A_n_, the m^6^A modification as a red circle, the m^6^A_m_ modification as a yellow circle, and the RNA degradation machinery as a red Pac-Man shape

m^6^A is the most abundant internal modification of messenger RNAs (mRNAs) and long non-coding RNAs (lncRNAs) (reviewed in [1]). Despite its initial discovery in 1974, the field of m^6^A biology remained largely unexplored for decades until the development of sequencing methods for mapping m^6^A modification in RNA species [2, 3]. However, quantifying m^6^A abundance at specific positions within individual transcripts remains a significant challenge, despite the development of numerous methodologies for mapping m^6^A distribution [4–7]. Currently, it is undoubtedly the most extensively studied RNA modification with the greatest therapeutic potential. Methylation at the N^6^ position of the target adenosine residue in RNA occurs co-transcriptionally by the methyltransferase complex composed of methyltransferase-like protein 3 (METTL3, also known as MT-A70) and methyltransferase-like protein 14 (METTL14) (Fig. 1) (reviewed in [8]). Although METTL14 lacks catalytic activity itself, it plays a crucial role in recognizing the DRACH sequence (D = A, G, or U; R = A or G; H = A, C, or U; A methylated adenosine) in RNA, facilitating methylation of the adenosine within this motif by the catalytic subunit, METTL3. This complex is further regulated by a multiprotein assembly including Wilms tumor 1-associated protein (WTAP), Vir-like m^6^A methyltransferase-associated (VIRMA, also known as KIAA1429), Cbl proto-oncogene like 1 (CBLL1), RNA-binding motif 15 (RBM15), and zinc finger CCCH-type containing 13 (ZC3H13) [8]. m^6^A is not randomly distributed, but rather targeted to specific regions within RNA molecules [8]. These enriched regions often occur near the stop codon, within the 3' untranslated region (3'UTR), or in particularly long exons. Furthermore, it has also been found in intronic regions of pre-mRNAs and long noncoding RNAs [8]. Several factors influence the precise location of m^6^A and thus its stoichiometry. These include the recruitment of the modifying complex by transcription factors, epigenetic modifications, or RNA-binding proteins to specific regions on the RNA. Additionally, the presence of protein complexes, such as the exon junction complex, can sterically hinder methylation in certain areas [9–11]. Notably, the methyltransferase METTL16, though primarily targeting U6 snRNA, can also recognize and modify certain mRNA and lncRNA substrates in a DRACH-independent manner [1]. The m^6^A modification can be removed by two "erasers", *ALKBH5* (alkB homolog 5) and *FTO* (fat-mass and obesity-associated protein) that belongs to the Fe^2+^ and 2-oxoglutarate (2OG)-dependent AlkB dioxygenase family [12, 13]. ALKBH5 exhibits high specificity for m^6^A in mRNA, while FTO acts on a broader range of modifications, including m^6^A_m_ at the cap, internal m^6^A_m_ within snRNAs, and m^1^A within tRNAs. FTO demethylase activity towards specific substrates is influenced by its subcellular localization and interaction with RNA binding proteins (Fig. 1) [14, 15].

m^6^A plays a crucial role in RNA metabolism, regulating virtually all stages of its expression, including transcription, splicing, export, stability, and translation (Fig. 2) (reviewed in [16]). The effects of m^6^A on gene expression are mediated by the binding of "reader" proteins Among the direct readers, proteins of the YTH domain family play a prominent role, including, YTHDC1, the only nuclear member, YTHDC2, YTHDF1, YTHDF2, and YTHDF3. These readers possess a domain that specifically recognizes the m^6^A modification regardless of the RNA sequence [8, 16]. YTHDC1 plays a part in transcriptional regulation and contributes to various nuclear RNA processing events, including alternative splicing, polyadenylation, and nuclear export [8, 16]. Additionally, it regulates the function of nuclear lncRNAs, such as *XIST* and *MALAT1*, and participates in the formation of nuclear RNA condensates [8, 16]. Despite their high sequence homology, the YTHDF proteins were initially ascribed distinct functions. The prevailing view held that YTHDF1 and YTHDF3 functioned as translation activators, while YTHDF2 was thought to promote mRNA degradation. New evidence suggests that YTHDF proteins function independently, potentially exhibiting redundancy in their ability to promote mRNA degradation [17, 18]. YTHDC2, the only RNA helicase-containing YTH protein, can also stimulate mRNA translation and decay by binding to m^6^A-modified transcripts. YTHDC2 plays a crucial role in the development and maturation of germ cells, particularly during spermatogenesis. However, recent research indicates that its function in spermatogenesis is independent of m^6^A modification [19]. Additionally, RNA-binding proteins, such as members of the IGF2BP family (IGFBP1, IGFBP2 and IGFBP3), FMRP, FXR1, and FXR2, exhibit enhanced binding to their consensus sequences when m^6^A is present, although the exact mechanism remains unclear. Furthermore, the presence of m^6^A can induce alterations in the local three-dimensional structure of RNA, a phenomenon termed "m^6^A switch," favouring or impeding the binding of RNA-binding proteins [8, 16]. Among these proteins are heterogeneous nuclear ribonucleoproteins (hnRNPs), including hnRNPA2B1, hnRNPC, and hnRNPG, as well as Ras GTPase-activating protein-binding protein 1 (G3BP1) and G3BP2. The binding of the latter to RNA is disfavoured by the presence of m^6^A [8, 16].

Given its critical role in gene expression, dysregulation of m^6^A levels or its reader proteins can have profound consequences for cellular function. This is reflected in the altered expression of m^6^A levels, or its readers observed in various cancers, where they contribute to tumour initiation, progression, treatment response, and resistance (reviewed in [20]). In this review, we describe the role of m^6^A in chronic myeloid leukemia (CML), a hematological malignancy characterized by the clonal expansion of myeloid cells [21], and discuss how selective inhibitors targeting the writers and erasers of this modification could be incorporated into therapeutic treatments of CML.

## Chronic myeloid leukemia (CML)

Hematological malignancies were among the first cancers where the oncogenic role of m^6^A was demonstrated, particularly acute myeloid leukemia (AML) [22–24]. Subsequently, m^6^A-linked oncogenic mechanisms have also been identified in CML [25–27]. CML is driven by the Philadelphia chromosome, resulting from a t(9;22)(q34;q11) translocation that creates the *BCR-ABL1* fusion gene. The *BCR-ABL1* fusion gene encodes a constitutively active tyrosine kinase, driving uncontrolled proliferation, impaired differentiation, and increased survival of myeloid progenitors by activating downstream pro-oncogenic signalling pathways including JAK/STAT, MAPK, and PI3K/AKT/mTOR pathways [21, 28, 29].

CML progresses through two distinct phases: the chronic phase and the acute phase, also known as blast crisis [30]. Some patients may have a transition between the two phases referred to as the accelerated phase, which is still lacking a precise biological definition. The chronic phase is the initial stage, characterized by relatively stable clinical manifestations and slower disease progression compared to later phases. Patients in chronic phase may be asymptomatic or have mild symptoms. Prior to the development of BCR-ABL1 tyrosine kinase inhibitors (TKIs), chronic phase CML patients invariably progressed to advanced stages of the disease, characterized by accelerated leukemic cell proliferation. This progression typically occurred within a median time frame of approximately 5 years. Therefore, timely diagnosis and initiation of targeted therapy with TKIs are paramount to achieving durable responses and preventing disease progression. The acute phase of CML is characterized by impaired differentiation and rapid proliferation of immature blasts (> 20% of bone marrow cells). While resembling AML in most cases, approximately 25% of patients exhibit pre-B lymphoblastic leukemia or, less frequently, T lymphoblastic transformation. Molecular and cytogenetic analyses may show additional chromosomal abnormalities and mutations in the epigenetic regulators such as ASXL1, DNMT3A, IDH1, and SETBP1 [31], indicative of clonal evolution and disease progression. This phase is associated with a dismal prognosis, with few treatment options available, and median survival of approximately 6 months. Early diagnosis and targeted therapy with TKIs are crucial for managing CML, with the advent of TKIs like imatinib dramatically transforming the prognosis for chronic phase patients, leading to deep molecular responses and prolonged survival. However, challenges remain, including persistence of leukemic stem cells that evade TKIs, development of TKI resistance, and long-term treatment-related toxicities. Aberrant m^6^A patterns have been implicated in the dysregulation of critical oncogenes and tumour suppressors in CML, thereby contributing to disease initiation, progression, and therapy resistance.

## m^6^A roles in CML

### Oncogenic roles for m^6^A regulators

METTL3 and METTL14 are upregulated in both primary chronic phase CML cells and established CML cell lines, including those derived from blast crisis [25]. Importantly, knocking down their expression leads to cell cycle arrest and decreased viability in both primary CML cells and CML cell lines [25]. Notably, this effect extends to imatinib-resistant CML cells, highlighting METTL3/METTL14 as a potential therapeutic target.

**Fig. 3:**
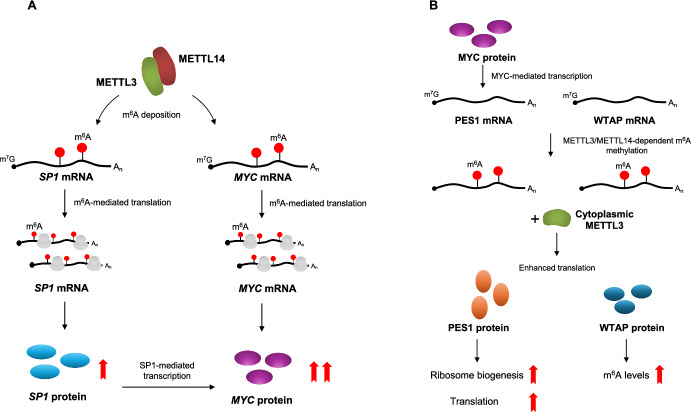
Oncogenic role for METTL3 in CML. **A** The METTL3-METTL14 complex promotes MYC expression through a two-pronged approach: indirectly by regulating SP1, a transcriptional activator of the MYC gene, and directly by enhancing MYC mRNA translation. **B** In CML cells, METTL3 is delocalized to the cytoplasm. Here, independent of its catalytic activity, METTL3 promotes the translation of PES1 and WTAP. PES1 is involved in ribosome biogenesis and cell cycle progression, while WTAP positively regulates the METTL3-METTL14 complex. Ultimately, the positive regulation of MYC and PES1 by METTL3 contributes to the aberrant protein translation that characterizes the leukemogenic activity of BCR-ABL1. Across all panels, RNA is depicted as a black line, the m^7^G at the 5' cap structure as a black dot, the poly(A) tail as A_n_, and the m^6^A modification as a red circle.

In CML, the interplay between global protein synthesis regulation and selective translation of specific mRNAs is crucial [32]. Similar to observations in AML, the METTL3-METTL14 complex promotes high levels of the *MYC* oncogene in CML [25]. *MYC* is one of the most common oncogenes in human cancers, exerting its growth-promoting effects primarily by enhancing ribosome biogenesis and mRNA translation [33]. The METTL3-METTL14 complex promotes *MYC* expression through a two-pronged approach: indirectly via SP1 regulation, a transcriptional activator of the *MYC* gene, and directly by enhancing *MYC* mRNA translation. Furthermore, CML exhibits cytoplasmic delocalization of METTL3. In this compartment, independent of its catalytic activity, METTL3 promotes the translation of PES1 and WTAP. PES1 is involved in ribosome biogenesis and cell cycle progression, while WTAP positively regulates the METTL3-METTL14 complex [24, 34]. Ultimately, the positive regulation of *MYC* and *PES1* by METTL3 contributes to the aberrant protein translation that characterizes the leukemogenic activity of BCR-ABL1 (Fig. 3).

**Fig. 4:**
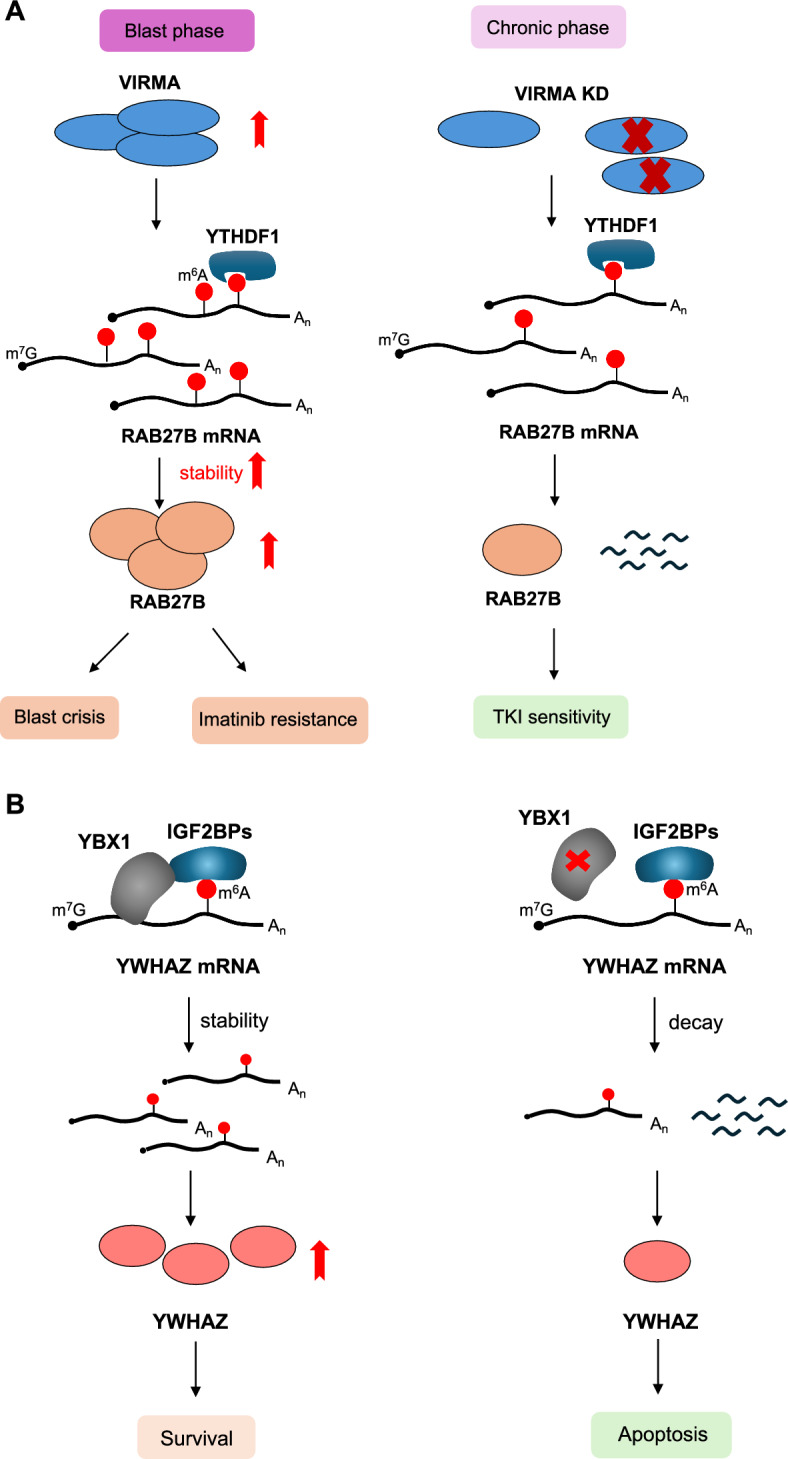
VIRMA and YBX1: mediators of m^6^A-driven leukemogenesis in CML. **A** VIRMA promotes m^6^A modifications, which, through the YTHDF1 reader protein, stabilize RAB27B mRNA. RAB27B is a protein involved in imatinib efflux from cells. Reduced RAB27B due to VIRMA knockdown increases intracellular imatinib levels, enhancing TKI sensitivity. **B** YBX1 cooperates with IGF2BP in CML cells to regulate the m^6^A-dependent stability of YWHAZ mRNA, which activates the PI3K/AKT/mTOR signaling pathway, a pathway important for cell survival. This mechanism likely contributes to YBX1's role in CML LSC survival. Across all panels, RNA is depicted as a black line, the m7G at the 5' cap structure as a black dot, the poly(A) tail as A_n_, and the m^6^A modification as a red circle.

*VIRMA* (*KIAA1429*), a regulator of the METTL3-METTL14 methyltransferase complex, is upregulated in acute-phase CML, contributing to elevated m^6^A levels [26]. Furthermore, VIRMA knockdown in CML cell lines recapitulates the effect of targeting the methyltransferase complex, leading to decreased proliferation and viability [26]. Importantly, VIRMA's m^6^A-stimulating action regulates *RAB27B* mRNA stability, a protein involved in imatinib efflux, through the YTHDF1 reader [26]. Reduced *RAB27B* levels due to VIRMA knockdown increase intracellular imatinib levels, consequently enhancing TKI sensitivity (Fig. 4A) [26].

An additional oncogenic mechanism mediated by m6A has recently emerged, centered on the RNA-binding protein *YBX1* [27]. Upregulated in CML patients, particularly those in the acute phase, YBX1 cooperates with IGF2BP protein in CML cell lines to regulate the m^6^A-dependent stability of *YWHAZ* mRNA, an activator of the PI3K/AKT/mTOR signaling pathway. This mechanism likely contributes to the role of *YBX1* in CML leukemia stem cells (LSCs) survival (Fig. 4B).

### Interplay between m^6^A and lncRNAs

**Fig. 5:**
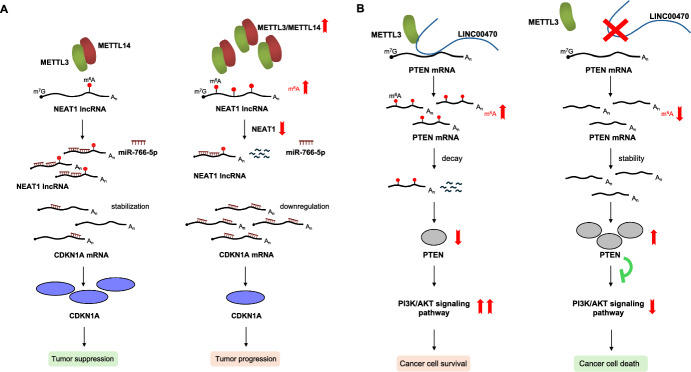
Regulation of lncRNA activity by m^6^A in CML. **A** NEAT1 expression progressively decreases in CML patients, with the sharpest decline occurring during the acute phase. This decrease coincides with elevated cellular m^6^A levels, potentially linked to CML-associated upregulation of the m^6^A methyltransferase complex. NEAT1 acts as a ceRNA for miR-766-5p, preventing it from downregulating the tumor suppressor CDKN1A. **B** The oncogenic LINC00470 guides the m^6^A methyltransferase METTL3 to the mRNA of the tumor suppressor PTEN. This interaction increases m^6^A levels of PTEN, leading to its degradation and subsequent activation of the PI3K/AKT signaling pathway, which promotes cell survival and potentially contributes to TKI resistance in CML

The lncRNA *NEAT1* (Nuclear paraspeckle assembly transcript 1) exhibits progressive downregulation in CML patients, with the most pronounced decrease observed in the acute phase [35]. *NEAT1* downregulation coincides with increased m^6^A levels due to CML-associated upregulation of the methyltransferase complex [24]. Notably, *NEAT1* overexpression in CML cell lines (K562 and KCL22) suppresses cellular viability, enhances apoptosis, and inhibits tumour growth in xenograft models [35]. Mechanistically, *NEAT1* acts as a competing endogenous RNA (ceRNA) for *miR-766-5p*, which downregulates the tumour suppressor *CDKN1A* (Yao 2021) (Fig. 5A). Furthermore, *NEAT1* overexpression in K562 cells negatively regulates the expression of the ABCG2 transporter protein, which is involved in the efflux of drugs from the cell and the development of drug resistance [36].

LncRNAs can also act as regulators of METTL3 activity. A prime example is *LINC00470*, which guides METTL3 to the mRNA of the tumour suppressor *PTEN* [37]. This interaction leads to an increase in m^6^A modification of *PTEN* mRNA, followed by its subsequent degradation. This results in the activation of the oncogenic PI3K/AKT signalling pathway, leading to increased AKT activity and its downstream targets (Fig. 5B). Furthermore, *LINC00470*-mediated PTEN reduction promotes autophagy via AKT hyperactivation promoting TKI resistance. Notably, decreasing *LINC00470* levels suppresses tumour growth in xenograft models, including those derived from imatinib-resistant K562 cells [37]. These findings suggest a potential role for *LINC00470* in TKI resistance mechanisms of CML. Interestingly, depletion of METTL3 in K562 cells abrogated the LINC00470-induced downregulation and degradation of *PTEN* mRNA and protein, restoring normal m^6^A modification levels in *PTEN* [37].

### m^6^A and DNA damage

**Fig. 6:**
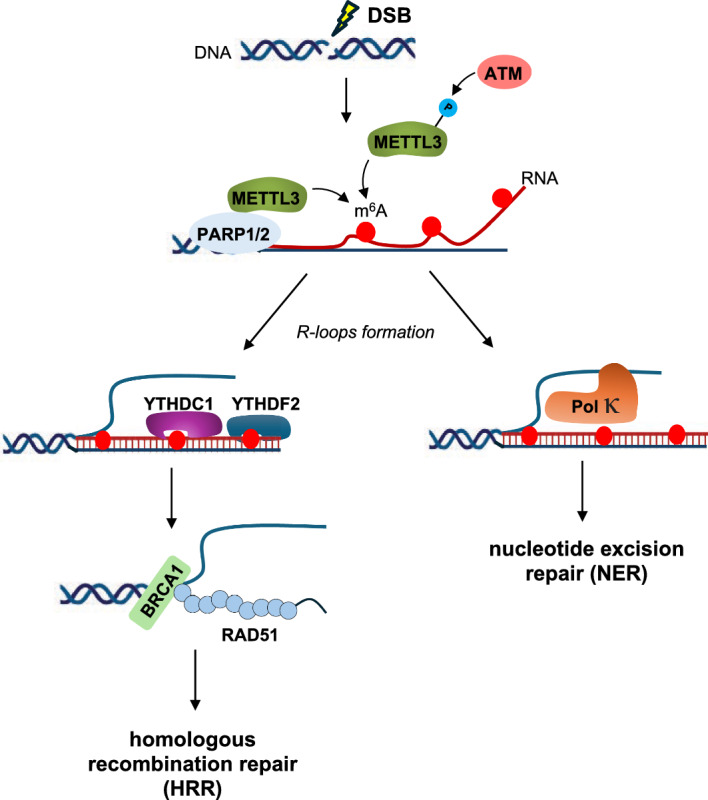
Regulation of DNA damage repair (DDR) by m^6^A modification. DNA damage triggers METTL3 recruitment to double-strand breaks (DSBs) via ATM phosphorylation or PARP1/2 interaction. METTL3 methylates RNA near the damage site, stabilizing these m6A-modified transcripts through YTHDC1 or YTHDF2 binding. Stabilized m^6^A-modified RNAs form R-loops with DNA, recruiting repair proteins like RAD51/BRCA1 for homologous recombination repair (HRR) or DNA Pol κ (Pol κ) for nucleotide excision repair (NER)

Genomic instability is a hallmark of TKI-resistant chronic phase CML, leading to disease relapse and/or malignant progression. This phenomenon likely arises from an aberrant cellular response to elevated DNA damage, including high levels of ROS-induced oxidative damage and compromised DNA damage repair (DDR) mechanisms [38]. Recent studies have shed light on a potential role for m^6^A in this context. Upon DNA damage, ataxia telangiectasia mutated (ATM) phosphorylates METTL3 promoting its localization at damaged sites. Here, METTL3 methylates nascent RNA transcribed at double-strand breaks (DSBs), potentially facilitating the formation of DNA-RNA hybrids (R-loops) and enhancing DDR (Fig. 6) [39, 40]. R-loops additionally, m^6^A modifications may regulate the expression of DNA repair genes, adding another layer of complexity to this interplay. Consequently, *METTL3* depletion sensitizes cancer cells to DNA damage-based therapies [39].

While a direct correlation between m^6^A and DNA damage in CML remains elusive, the emerging link between m^6^A signalling and DDR pathways holds promise for novel therapeutic strategies. This is particularly relevant because *BCR-ABL1*, the driver oncogene in CML, downregulates *BRCA1*, a critical protein for homologous recombination repair (HRR) [41]. Consequently, BCR-ABL1 activity leads to increased DNA damage. PARP inhibitors (PARPi), such as olaparib, exploit this vulnerability by further inhibiting base excision repair [41]. PARP1 plays a crucial role in repairing single-strand breaks (SSBs) in DNA. It detects and binds to damaged DNA, facilitating the recruitment of other repair proteins to the site of damage. These unrepaired SSBs can convert into single-ended DSBs during replication, a type of damage that is predominantly repaired by BRCA1-mediated HRR [41]. In HRR-deficient cells, such as CML, PARPi treatment in combination with METTL3 inactivation may induce a "synthetic lethality" effect, where the combined inhibition of two DNA repair pathways becomes lethal to the cancer cells. Notably, olaparib treatment has been shown to decrease the proliferation of CML cells in vitro and improve survival in a BCR-ABL1-dependent leukemia mouse model, highlighting its therapeutic potential in CML [42, 43]. Further research is warranted to explore the potential therapeutic effects of combining PARPi with METTL3 inhibitors in CML cells not responding to TKIs.

### m^6^A and TKI resistance

Point mutations that reduce drug-binding affinity are a major cause of TKI resistance, although second and third generation TKIs are often designed to overcome these mutations (reviewed in [31]). Currently, five distinct TKIs against BCR-ABL1 fusion are available for the treatment of the chronic phase of CML: imatinib, dasatinib, nilotinib, bosutinib, ponatinib and asciminib. However, switching TKIs in resistant patients might inadvertently promote the emergence of complex mutations that render the cancer cells insensitive to most, if not all, available TKIs. Additionally, a subset of patients develops TKI resistance despite lacking identifiable resistance mutations. Patients harbouring TKI resistance mutations exhibit an increased propensity to transition into the blast crisis.

**Fig. 7:**
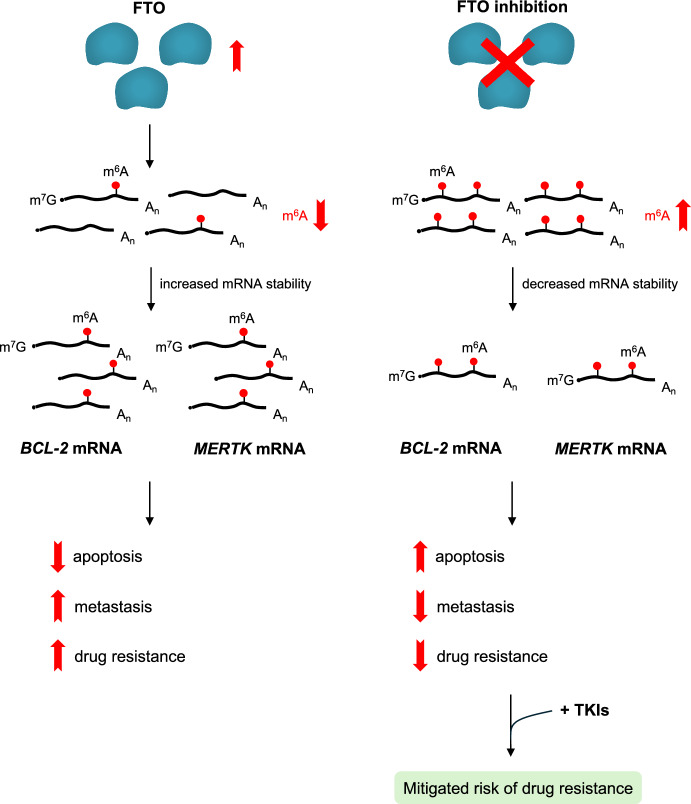
m^6^A and TKI resistance. TKI-resistant cells often exhibit upregulation of FTO and decreased m^6^A methylation levels. This reduction in cellular m^6^A levels leads to increased stability and expression of mRNAs encoding proteins involved in cell proliferation and survival, such as the MERTK and BCL-2 proteins. Consequently, TKI-resistant cells experience reduced apoptosis, increased metastasis, and ultimately, resistance to TKI therapy. Conversely, strategies that decrease FTO expression or inhibit its catalytic activity can restore m^6^A levels, potentially leading to decreased mRNA stability of these pro-survival genes and a reduced risk of TKI resistance. Across all panels, RNA is depicted as a black line, the m^7^G at the 5' cap structure as a black dot, the poly(A) tail as A_n_, and the m^6^A modification as a red circle.

In CML cell lines, a decrease in global m^6^A levels in mRNAs has been linked to TKI resistance against imatinib and nilotinib [44]. *FTO* upregulation in resistant cells is thought to cause a reduction in m^6^A levels, leading to subsequent upregulation of mRNA expression for genes involved in cellular proliferation and survival, particularly the myeloid epithelial reproductive tyrosine kinase receptor (*MERTK*) and B-cell lymphoma 2 (*BCL-2*) (Fig. 7). Both *MERTK* and *BCL-2* are well-established contributors to reduced apoptosis, increased metastasis, and drug resistance. Notably, resistance to apoptosis is a hallmark of CML LSCs. This link is further supported by similar findings in primary CML cells with induced nilotinib resistance. These cells also displayed increased FTO levels and decreased m^6^A levels in the *MERTK* and *BCL-2* mRNA. This finding suggests that FTO inhibition could be employed in combination with TKI therapy to mitigate the risk of resistance in CML. Intriguingly, elevated FTO expression in AML is associated with resistance to both TKI therapy and chemotherapy. This finding suggests that FTO inhibition could be a valuable strategy to overcome multidrug resistance and prevent disease relapse in leukemia.

### m^6^A as therapeutic target

Through a combination of virtual screening and rational design researchers have developed a repertoire of potent and selective m^6^A machinery inhibitors. Notably, some of these inhibitors exhibit high efficacy in pre-clinical models of AML. The first inhibitors of m^6^A regulators were developed against the enzyme FTO, whose crystal structure has been known since 2010 [45]. While elevated m^6^A levels are generally considered oncogenic, the m^6^A eraser FTO has emerged as a promising therapeutic target in certain AML subtypes and, notably, in acquired TKI resistance in both AML and CML [44]. The first selective FTO inhibitors were derived from the meclofenamic acid molecule. Particularly, FB23-2 exhibited potent antiproliferative activity against AML cells both in vitro and in vivo, but still with a IC50 in the micromolar range. Structure-based virtual screening recently identified CS2 (formerly brequinar) as a potent inhibitor of FTO demethylase activity, demonstrating potent antileukemic activity in vitro and in vivo with a nanomolar IC50. However, CS2 also potently inhibits human dihydroorotate dehydrogenase (*h*DHODH), an enzyme crucial for de novo uridine synthesis, and targeting *h*DHODH itself has independent antileukemic activity. This raises the question of whether CS2's primary mechanism in leukemia involves hDHODH or FTO inhibition. Notably, CML cells are particularly sensitive to *h*DHODH inhibition [46]. Therefore, CS2 could offer a dual benefit: antiproliferative activity through *h*DHODH inhibition and potential mitigation of resistance via FTO inhibition. Furthermore, the activity of FTO extends beyond demethylating m^6^A on mRNA. Its roles in demethylating other RNA substrates, including m^6^A on snRNAs, cap-associated m^6^A_m_ on mRNAs and snRNAs, and m^1^A on tRNA, which have been largely neglected in recent studies, warrant further investigation to comprehensively understand the biological effect of FTO inhibition.

Given the oncogenic role of METTL3 in various malignancies, several inhibitors have been developed (reviewed in [47]). Early attempts to develop METTL3 inhibitors focused on designing competitive inhibitors of its cofactor S-adenosylmethionine (SAM). These inhibitors are therefore capable of suppressing METTL3 catalytic activity when in complex with METTL14 but not the independent catalytic action that has been described for the protein when localized in the cytoplasm. Among these, UZH2 and STM2457 stand out as the most selective and effective inhibitors in preclinical leukemia models. These inhibitors have demonstrated efficacy in a broad spectrum of acute myeloid leukemias, and mice and human PDX AML models, suggesting their potential effectiveness in CML blast crisis and TKI-resistant cells, where METTL3 knockdown exhibits a robust antiproliferative effect [25]. However, the cytoplasmic delocalization of METTL3 in CML, where it exerts an oncogenic role independent of its catalytic activity, could potentially limit the efficacy of these inhibitors. The development of degradative inhibitors, such as proteolysis targeting chimeras (PROTACs), like those recently derived from the UZH2 inhibitor, holds promise as a therapeutic strategy for CML patients in blast crisis.

Furthermore, inhibitors targeting FTO and METTL3 could be employed in combination therapy with clinically approved molecules. For instance, considering that FTO upregulation of MERTK and BCL2 contributes to CML TKI resistance, a combination of catalytic FTO inhibitors with MERTK-targeting monoclonal antibodies or selective BCL2 inhibitors like venetoclax could be explored. Similarly, given the recent demonstration that METTL3 catalytic inhibition sensitizes tumor cells to genotoxic agents, such as the PARPi olaparib [48], METTL3 inhibitors could be utilized to enhance the efficacy of these agents to eliminate LSCs and to induce synthetic lethality in CML patients that do not respond to standard TKI-based therapy.

## Future directions

Understanding the role of m^6^A regulators in CML holds immense potential for novel therapeutic strategies. As discussed, even if CML and AML are characterized by different genetic alterations, both diseases can progress to a state of rapid blast cell proliferation, potentially sharing overlapping dysregulation in cellular pathways. Therefore, investigating m^6^A regulators and pathways implicated in AML is crucial to gain insights into their potential role in CML, particularly in blast crisis. Here, we explore key areas for future investigation. While the m^6^A demethylase ALKBH5 has been implicated in promoting leukemogenesis and poor prognosis in AML [49], its role in CML remains largely unexplored. Future studies should focus on understanding whether ALKBH5 expression or activity is dysregulated in CML patients compared to healthy controls. Additionally, investigating the impact of ALKBH5 activity on the response of CML cells to current therapies or its influence on leukemia development could shed light on its potential role in the disease process. These investigations hold promise for revealing whether targeting ALKBH5 could be a viable therapeutic strategy for CML. Furthermore, the role of m^6^A readers in CML remains largely unelucidated. YTHDF2 is upregulated in AML samples [50]. The protein is essential for LSC self-renewal, and its depletion compromises the ability of LSCs to expand and thus propagate AML. Therein, it will be interesting to analyze its downregulation in CML. In AML, the nuclear m6A reader YTHDC1 promotes the formation of m^6^A-containing mRNA condensates, stabilizing oncogenic transcripts like *MYC* mRNA and promoting cancer cell survival [51]. *MYC* also plays a critical role in CML, suggesting that this mechanism might be relevant in this disease as well. Here, further investigation is warranted to determine if YTHDC1 and m^6^A-mediated mRNA stabilization contribute to CML pathogenesis. Similarly, Insulin-like growth factor-2 mRNA-binding proteins (IGF2BP1/2/3) have been found to be overexpressed in AML, and to regulate the stability of specific mRNAs, including *MYC*, in an m^6^A-dependent manner to promote tumor progression [52–54]. Also, the functions of the newly identified m^6^A reader proteins, such as PRRC2A/B and FXR1, in the context of leukemia remain largely unexplored. Future investigations should explore the expression patterns of these readers in CML cells compared to healthy controls and their functional consequences in m^6^A-regulated pathways relevant to CML development and progression. Additionally, the potential of manipulating reader function (e.g., through small molecule inhibitors) as a viable therapeutic strategy for CML warrants further exploration.

Finally, the intricate interplay between m^6^A RNA methylation and the unfolded protein response (UPR) pathway remains largely unexplored in CML. While the UPR pathway is a known therapeutic target in CML [55, 56], the interplay between UPR and m^6^A RNA methylation remains poorly understood in this context. Existing research suggests a complex and cell-type specific role for m^6^A regulators in the ER stress response. Studies in mouse liver cells and other cell types demonstrate opposing effects of METTL3 and METTL14 on ER stress, highlighting the context-dependent nature of this interaction [57–59]. Similarly, studies in breast cancer reveal opposing functions of m^6^A regulators YTHDF2 and VIRMA [60, 61]. YTHDF2 reader downregulation induces ER stress and apoptosis, while VIRMA overexpression increases UPR regulator expression under stress, possibly through m^6^A modification. Interestingly, ER stress can also influence m^6^A machinery by elevating METTL3/METTL14 levels, promoting mRNA stability for proteins involved in ER-phagy (a specific form of autophagy targeting ER components) [62]. Importantly, knocking out METTL3/METTL14 sensitizes breast cancer cells to ER stress-inducing drugs [62]. These findings suggest a complex loop between m^6^A and ER stress, where each component can influence the other. Furthermore, these observations suggest that leukemia cells with elevated m^6^A levels might be more sensitive to UPR inducers. However, the molecular mechanisms linking ER stress-mediated m^6^A regulation and its role in the UPR pathway remain largely unexplored in leukemia.

By addressing these future directions, researchers can gain a deeper understanding of the m^6^A landscape in CML.

## Conclusions

In conclusion, the burgeoning field of m^6^A RNA modification provides a compelling framework to elucidate the molecular underpinnings of CML pathogenesis, progression and resistance. Overall, targeting the m6A machinery offers a novel and promising therapeutic approach for CML, with the potential to improve treatment outcomes, particularly in patients with advanced disease or TKI resistance. By integrating the knowledge of m^6^A biology with ongoing therapeutic advancements, we can strive for more effective and personalized treatment strategies for CML patients.

Across all panels, RNA is depicted as a black line, the m^7^G at the 5' cap structure as a black dot, the poly(A) tail as A_n_, and the m^6^A modification as a red circle.

## Data Availability

Not applicable.
